# Composite Gel Polymer Electrolyte for High‐Performance Flexible Zinc‐Air Batteries

**DOI:** 10.1002/smll.202408015

**Published:** 2024-11-13

**Authors:** Yifan Liu, Denise Bildan, Xiangqun Zhuge, Tong Liu, Haoyang Zhong, Zhihong Luo, Hanhui Lei, Kun Luo, Yurong Ren, Maryam Bayati, Xiaoteng Liu

**Affiliations:** ^1^ Jiangsu Province Engineering Research Centre of Intelligent Manufacturing Technology for the New Energy Vehicle Power Battery School of Materials Science and Engineering Changzhou University Changzhou 213164 P R China; ^2^ Department of Mechanical and Construction Engineering Faculty of Engineering and Environment Northumbria University Newcastle upon Tyne NE1 8ST UK; ^3^ School of Materials Science and Biomedical Engineering University of Wisconsin‐Eau Claire 105 Garfield Avenue Eau Claire WI 54702‐4004 USA; ^4^ College of Materials Science and Engineering Guilin University of Technology Guilin 541004 P R China

**Keywords:** composite, flexible zinc‐air battery, gel polymer electrolyte, mesoporous silica

## Abstract

Enhancing ionic conductivity and electrolyte uptake is of significance for gel polymer electrolytes (GPEs) for flexible zinc‐air batteries (FZABs). Herein, a composite mesoporous silica/polyacrylamide (5 wt.% mPAM) GPE is constructed with comparable ionic conductivity to aqueous electrolytes, where the ionic conductivity is up to 337 mS cm^−1^, and the weight loss after exposing in air 72 h is less than 18%, owing to the excellent electrolyte uptake and continuous ion migration network provided by the mesoporous silica fillers. When used as a quasi‐solid‐electrolyte, the rechargeable FZAB exhibited high electrochemical performance and structural stability, where the peak power density is up to 162.8 mW cm^−2^, and the initial charge–discharge potential gap is as low as 0.62 V, resulting in a long lifespan exceeding 110 h, showcasing the combination of high durability, cost‐effectiveness and easy production for practical applications.

## Graphical abstract

Incorporation with mesoporous silica (mSiO_2_) significantly improves the ionic conductivity, electrolyte uptake, and retention of PAM‐based gel polymer electrolytes (GPEs), where the hydrophilic surface and mesopores (ca. 3.6 nm) of the mSiO_2_ can provide extra space to retain the electrolyte and additional pathways to facilitate ion transport, showcasing the combination of high durability, structural stability, cost‐effectiveness, and easy production. The assembled flexible zinc‐air battery with the optimized 5 wt.% mPAM GPE exhibits a high peak power density of up to 162.8 mW cm^−2^, together with a long lifespan exceeding 110 h.

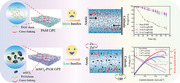

## 1 Introduction

The development of energy storage devices has become a critical demand for lightweight, flexible, and wearable technologies.^[^ ^1^, ^2^, ^3^ ^]^ Flexible zinc‐air batteries (FZABs) have garnered growing attention due to their high energy density (1086 Wh kg^−1^), inherent safety, low cost, and environmental friendliness,^[^ ^4^, ^5^, ^6^, ^7^ ^]^ compared to ordinary lithium‐ion batteries. Gel polymer electrolytes (GPEs) are positioned between liquid and solid electrolytes, combining the advantages of excellent safety, interfacial stability, and flexibility.^[^ ^8^ ^]^ These characteristics make GPEs particularly suitable for use in wearable and medical electronic devices.

As one of the commonly used GPEs, polyvinyl alcohol (PVA) exhibits excellent chemical stability, electrochemical inertness, durability, non‐toxicity, and simplicity of preparation.^[^ ^9^ ^]^ Fan^[^ ^10^ ^]^ et al prepared a PVA‐KOH GPE by absorbing the KOH solution, which allowed a FZAB to cycle for 12 h. Meanwhile, they also observed that the cycle life was shortened to 2 h if the FZAB was pre‐exposed to air for 8 h, and the peak power density decreased from 51 to 24 mW cm^−2^. Using a pore‐forming agent polyethylene glycol (PEG)^[^ ^11^ ^]^ was reported to significantly enhance the ionic conductivity of PVA GPE from 37 to 58 mS cm^−1^, and the corresponding power density of the FZABs was improved from 39 to 63 mW cm^−2^. However, challenges persist in the conventional PVA‐KOH GPE system as follows: 1) low concentration of KOH solution (<2 mol L^−1^) is allowed and limits the ionic conductivity (10^−4^–10^−3^ S cm^−1^);^[^ ^12^, ^13^ ^]^ 2) tight molecular cross‐linking leads to poor electrolyte uptake,^[^ ^11^, ^13^ ^]^ resulting in serious electrolyte loss in long‐term cycling of the semi‐open FZABs.^[^ ^14^, ^15^ ^]^

Recently, polyacrylamide (PAM) has attracted growing interest for its high ionic conductivity, excellent electrolyte uptake, and interfacial contact to electrodes for rechargeable FZABs.^[^ ^16^, ^17^, ^18^ ^]^ In addition, PAM is also safe enough to apply in cosmetics, food processing, and biomedicine.^[^ ^19^ ^]^ Tan^[^ ^16^ ^]^ et al synthesized PAM through UV irradiation and achieved remarkable ionic conductivity of up to 0.33 S cm^−1^. When used in a FZAB, the power density reached 39 mW cm^−2^ accompanied with stable charge–discharge cycling for more than 50 h. Miao^[^ ^17^ ^]^ et al applied a prepared oxygen reduction catalyst (MnO_2_/NRGO_‐Urea_)to PAM FZABs, achieving a corresponding power density of 105 mW cm^−2^, with a charge‐discharge cycle life lasting up to 140 h at a current density of 5 mA cm^−2^. Polyacrylic acid (PAA), polyacrylate (PAA‐Na), and tetraethylammonium hydroxide (TEAOH) are also used as alternative alkaline GPEs for FZABs.^[^ ^20^, ^21^, ^22^ ^]^

Solid‐state electrolytes should exhibit high ionic conductivity, a broad electrochemical window, enough mechanical strength, and great electrode compatibility.^[^ ^23^ ^]^ Thereby, inorganic fillers have drawn extensive attention to improving the ionic conductivity and interfacial contact to electrodes of GPEs.^[^ ^24^, ^25^ ^]^ Three major effects of these fillers have been identified, i.e., barrier effect, intrinsic dielectric constant effect, and water retention effect.^[^ ^26^ ^]^ Inorganic fillers can reduce the aggregates and crystallinity of polymer and increase the number of polymer chain segments,^[^ ^27^ ^]^ facilitating the dissociation of metal ions or establishing new ion conductive channels. Shang^[^ ^28^ ^]^ et al introduced silica (SiO_2_) particles ranging from 5 to 15 µm into PAA matrix, which led to the loose gel skeleton structure and increase of ion transport channels. Moreover, unreacted SiO_2_ served as an additional water retention agent and plasticizer. The latest study turned to concentrate on the water uptake capability of porous inorganic fillers. Rieth^[^ ^29^ ^]^ et al reported that the introduction of metal‐organic frameworks resulted in a significant increase in water retention, owing to the thermodynamic favorability of water pore‐filling depends more strongly on the pore diameter and the interface between water in the center of the pore and water bound to the pore walls.

In this study, we designed and synthesized a GPE of mesoporous silica (mSiO_2_)/PAM (mPAM) composite, based on the polymer substrates (PAM and PVA) and loading amount of mSiO_2_. The optimized GPE (5 wt.% mPAM) exhibits the excellent ionic conductivity, electrolyte retention, and mechanical ability, and significantly enhances the electrochemical performance of FZABs, where the peak power density was up to 162.8 mW cm^−2^, and the initial charge‐discharge potential gap was as low as 0.62 V, resulted in a long lifespan exceeding 110 h. This innovation offers a new pathway for developing robust free‐standing GPEs for high‐performance FZABs, with high ionic conductivity, superior electrolyte uptake and retention, excellent mechanical properties, and cost‐effectiveness.

## 2 Results and Discussion

### 2.1 Characterizations of mPAM GPEs

**Figure 1:**
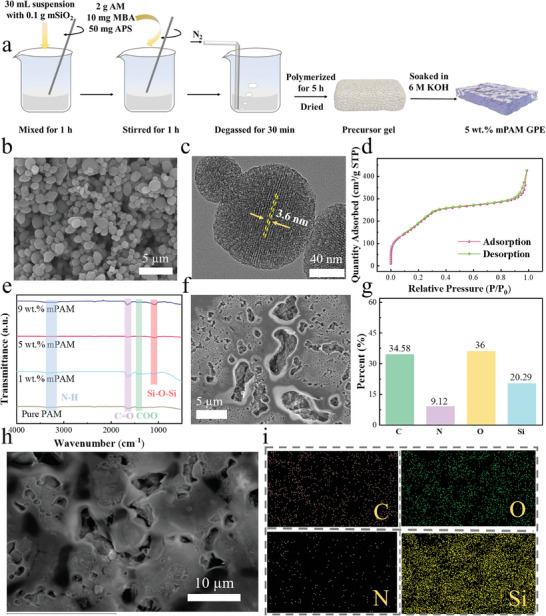
Schematic diagram for the synthesis of the 5 wt.% mPAM GPE a); SEM b) and HRTEM c) and N_2_ absorption/desorption isotherm d) of the mSiO_2_ fillers; FTIR e) of the pure PAM, 1 wt.% mPAM, 5 wt.% mPAM and 9 wt.% mPAM; SEM f) and EDX analysis g) of the 5 wt.% mPAM; h,i) C, O, N, Si elemental mapping of the 5 wt.% mPAM GPE.

**Figure** **1**b and S1b(Supporting Information) present SEM images of the as‐synthesized mSiO_2_ nanoparticles prior to the synthesis of mPAM GPEs. From the scanning images, it can be observed that the majority of mSiO_2_ particles exhibit an elliptical shape, with a minority showing a spherical shape. Figure S1 (Supporting Information)c further illustrates that the average particle size of mSiO_2_ nanoparticles is 175 ± 10 nm (N = 100). Figure S1d and 1c (Supporting Information) depict high‐resolution transmission electron microscope (HRTEM) images of mSiO_2_ particles, and the close‐packed parallel mesopores are displayed. Figure S1e (Supporting Information) shows the FTIR of mSiO_2_ nanoparticles before and after calcination at 600 °C, revealing the presence of Si─O─Si antisymmetric stretching peaks at 1065 cm^−1^.^[^ ^30^ ^]^ However, after calcination, the stretching vibration peaks corresponding to the ─CH_2_‐ of CTAB at 2850 and 2918 cm^−1^, as well as the stretching vibration peak corresponding to H─C─H of CTAB at 1482 cm^−1^, disappear, indicating the removal of the template agent CTAB in line with previous literature.^[^ ^31^ ^]^ Figure S1f (Supporting Information) presents the small‐angle XRD pattern of mSiO_2_ nanoparticles, where the characteristic peaks appear at 2–3° and 4–5°, indicative of the hexagonal arrangement of mesopores,^[^ ^32^ ^]^ and the wide peak at 23° in the inset suggests the amorphous nature of mSiO_2_ nanoparticles.^[^ ^33^ ^]^ Figure 1d exhibits a typical type IV isotherm in nitrogen adsorption/desorption experiments, and the corresponding specific surface area is determined as 571.57 m^2^ g^−1^, with an average pore diameter of 3.6 nm estimated by the BJH model^[^ ^34^, ^35^ ^]^ (see Figure S1 g, Supporting Information).

Figure 1a illustrates the synthesis process of the mPAM GPEs, and the synthetic reaction is shown in Figure S2 (Supporting Information). FTIR analysis in Figure 1e displays two common peaks at ≈3300 and 1600 cm^−1^ for the pure PAM and composite mPAM GPEs filled with 1, 5, and 9 wt.% of mSiO_2_, corresponding to the stretching vibrations of N─H and C═O in AM, respectively. The peak at 1400 cm^−1^ corresponds to COO^−^,^[^ ^16^ ^]^ while the peak at 1180 cm^−1^ is assigned to the asymmetric stretching of Si─O─Si bonds,^[^ ^36^ ^]^ and a broad absorption peak appears in the range of 3200–3500 cm^−1^, primarily attributed to the stretching vibrations of N─H bond, indicating the presence of both mSiO_2_ and PAM in the 1, 5 and 9 wt.% mPAM GPEs.^[^ ^37^ ^]^ It can be predicted that the binding between the mSiO_2_ filler and PAM matrix is also strong by the hydrogen bonding between the hydroxyl group of mSiO_2_ and the PAM molecule.

Figure 1f and inset illustrate the SEM and EDX elemental mapping of 5 wt.% mPAM GPE. Based on the Si element distribution from the EDX analysis, it is evident that the pore of mPAM enhances ionic conductivity and electrolyte uptake, while the uniform dispersion of mSiO_2_ can be expected to further improve performance. As a reference, Figure S3a (Supporting Information) displays the smooth surface of Pure PAM without pores, and Figure S3b and S3c (Supporting Information) manifests the surface morphology of 1 mPAM and 9 wt.% mPAM GPEs with pores, which clearly suggests that the PAM becomes too little to cover mSiO_2_ fillers if the content is as high as 9 wt.%.

### 2.2 FZABs with Composite mPAM GPEs

**Figure 2:**
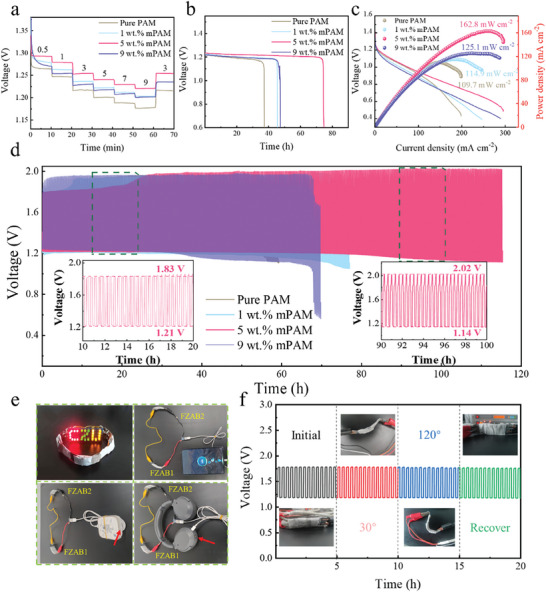
Rate performance a), full‐discharge capacity at 3 mA cm^−2^ b), discharge polarization and power density curves c); and cyclability at 3 mA cm^−2^ d) of the FZABs with the pure PAM, 1 wt.% mPAM, 5 wt.% mPAM and 9 wt.% mPAM GPEs, the inset highlights the cycles with the 5 wt.% mPAM GPE at the 10–20 h and 90–100 h intervals; illustration of charging various electronic devices with the two‐FZAB pack e) and flexibility and stability of the FZAB cycling at different bending conditions f).

FZABs were constructed by a GPEs‐based “sandwich” battery model, as depicted in Figure S4 (Supporting Information). Battery testing results manifest a notable improvement by using the mPAM GPE in comparison to the pure PAM GPE. In terms of pulse discharge performance (see **Figure** **2**a), the FZAB with 5 wt.% mPAM GPE exhibits the voltage of 1.29 V at 0.5 mA cm^−2^ and 1.22 V at 9 mA cm^−2^, i.e., the voltage drop is only 0.07 V as the current density increased for 18 times, however, the voltage varies much larger for the pure PAM, 1 wt.% mPAM and 9 wt.% mPAM GPEs. Figure 2b displays the consistent discharge performance at a constant rate of 3 mA cm^−2^, where the 5 wt.% mPAM GPE allows the flexible cell to operate for ≈75 h, significantly longer than the 37, 46, and 47 h for the pure PAM, 1 wt.% mPAM and 9 wt.% mPAM GPEs, respectively.

Figure 2c shows the polarization and power density profiles, in which the cell with the 5 wt.% mPAM exhibits a maximum current density of 295.4 mA cm^−2^ and a peak power density of up to 162.8 mW cm^−2^, much better than the batteries with the pure PAM (198.7 mA cm^−2^, 109.7 mW cm^−2^), 1 wt.% mPAM (246.3 mA cm^−2^, 114.9 mW cm^−2^) and 9 wt.% mPAM (287.6 mA cm^−2^, 125.1 mW cm^−2^) GPEs, which is the largest power density of FZABs (ref. Table S1, Supporting Information), to the best of our knowledge.

Figure 2d presents the cyclic performance of the FZABs of PAM‐based GPEs at 3 mA cm^−2^. The FZAB with the 5 wt.% mPAM GPE presents outstanding rechargeable cycling for up to 336 cycles (or 115 h) because the charge/discharge voltage gap is the smallest among the FZABs with the pure PAM, 1 wt.% mPAM and 9 wt.% mPAM GPEs, where the inset of Figure 2d highlights the voltage variation ranging from 10 h to 20 h (0.62 V) and from 90 h to 100 h (0.88 V) of charge/discharge cycling. To test the electrolyte retention on the performance of FZABs, the pure PAM, 1 wt.% mPAM, 5 wt.% mPAM and 9 wt.% mPAM GPEs were allowed to be exposed in open air for three days and then were used to assemble FZABs. Figure S5a (Supporting Information) shows that the rate performance with the 5 wt.% mPAM electrolyte just slightly decreased, and the others apparently lowered down. Figure S5b (Supporting Information) illustrates that the FZAB with the 5 wt.% mPAM GPE could discharge for almost 51 h, significantly longer than other FZABs with the pure PAM (13 h), 1 wt.% mPAM (27 h) and 9 wt.% mPAM (35 h). Figure S5c (Supporting Information) displays that the FZAB with the 5 wt.% mPAM exhibit a maximum current density of 146.8 mA cm^−2^ and a peak power density of 118.8 mW cm^−2^, higher than the pure PAM (121.3 mA cm^−2^, 74.2 mW cm^−2^), 1 wt.% mPAM (118.9 mA cm^−2^, 76.7 mW cm^−2^) and 9 wt.% mPAM (110.0 mA cm^−2^, 81.29 mW cm^−2^). Figure S5d (Supporting Information) illustrates that the FZAB with the 5 wt.% mPAM GPE remains good cyclic stability for up to 65 h, and the insets highlight the intervals of 10–20 and 50–60 h.

When two FZABs were connected in series, led to a notable open‐circuit voltage of 2.95 V (see Figure S6a, Supporting Information). Thereby, the FZABs assembled with the 5 wt.% mPAM GPE shown in Figure 2e could not only light up the LED sign of “CZU” representing Changzhou University but also were able to charge other electronic products such as mobile phones, headphones, Bluetooth earphones, and tablets for no less than 1 h, highlighting the FZAB's impressive energy density and durability. In addition, Video S1 (Supporting Information) demonstrates that a single FZAB could power a small fan. Figure 2f illustrates the superior properties in charge/discharge cycling with the shape change of the battery, in which the FZAB demonstrates impressive operation stability at the bending angles of ≈0°, 30° and 120°.

**Figure 3:**
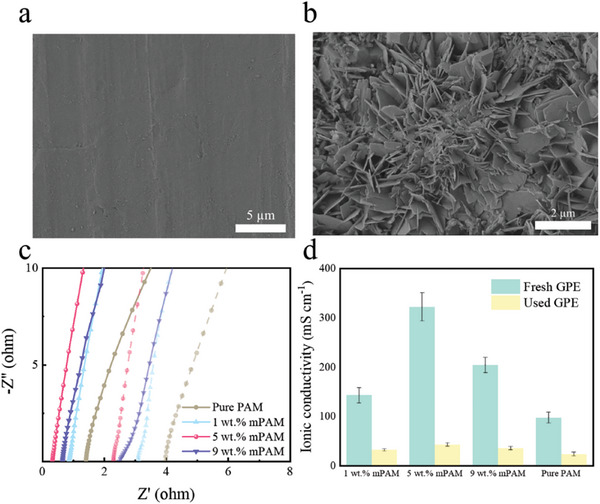
SEM images of the fresh a) and used b) zinc anodes the failed FZAB, and Nyquist plots of the used GPEs c) and variation of ionic conductivity for the fresh and used GPEs d).

**Figure** **3** shows the analysis of the zinc anode and 5 wt.% mPAM GPE in a failure FZAB. SEM analysis illustrates that a large amount of zinc dendrites are visible on the used zinc anode (Figure 3b), where the pristine zinc sheet has a smooth surface (Figure 3a). On the other hand, Figure 3c,d show the EIS analysis on the used 5 wt.% mPAM GPE (42.62 mS cm^−1^), and compares its ionic conductivity with the pristine 5 wt.% mPAM GPE (322 mS cm^−1^). The sharp decrease of ionic conductivity causes the increase of inner electric resistance of the FZAB, and significantly widens the charge/discharge potential gap, leading to quick battery failure. Similarly, the ionic conductivity decreases sharply in other FZABs with the pure PAM, 1 wt.% mPAM, and 9 wt.% mPAM GPEs as well, where the ion conductivities drop down to 23.4, 31.65, and 35.42 mS cm^−1^, respectively. This reminds us that the degradation of GPEs plays a more important role than the zinc anode on FZABs’ failure.

### 2.3 Effect of GPEs on Battery Performance

The properties of GPEs, such as ionic conductivity, electrolyte uptake and retention, mechanical property, and interfacial compatibility to electrodes, have immense effects on the application of FZABs, which involve in the polymer matrix and filler of the composite 5 wt.% mPAM GPE.

**Figure 4:**
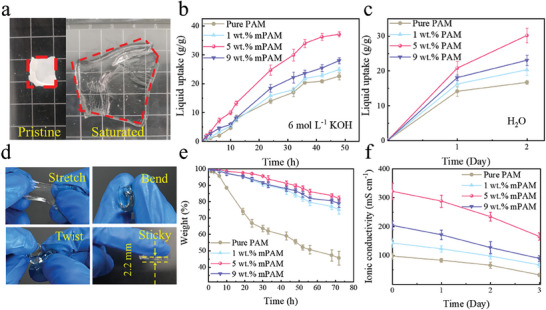
Dry and electrolyte saturated 5 wt.% mPAM GPE a); liquid uptake ratios in 6 mol L^−1^ KOH b) and deionized water c) of the pure PAM, 1 wt.% mPAM, 5 wt.% mPAM and 9 wt.% mPAM GPEs; stretching, torsion, bending, and sticky features of the 5 wt.% mPAM GPE d); electrolyte retention h) and ionic conductivity i) of the pure PAM, 1 wt.% mPAM, 5 wt.% mPAM and 9 wt.% mPAM GPEs.

In the aspect of electrolyte uptake, **Figure** **4**a displays that the composite 5 wt.% mPAM GPE significantly swelled when absorbed electrolytes, turning from a white‐colored membrane to a dramatically expanded transparent film. Similarly, Figure S7 (Supporting Information) demonstrates the same behavior for the pure PAM, 1 wt.% mPAM, and 9 wt.% mPAM GPEs. Figure 4b,c illustrate the liquid uptake ratios in 6 mol L^−1^ KOH solution and deionized water, in which the 5 wt.% mPAM membrane could absorb 37 g g^−1^ KOH solution and 30 g g^−1^ water. In contrast, less amounts of electrolyte were held by the pure PAM (22 g g^−1^ KOH solution and 17 g g^−1^ water), 1 wt.% mPAM (25 g g^−1^ KOH solution and 20 g g^−1^ water) and 9 wt.% mPAM GPEs (28 g g^−1^ KOH solution and 23 g g^−1^ water), respectively. As shown in Figure 4d, the 5 wt.% mPAM GPE demonstrates excellent stretching, bending, and twisting properties, and is sticky to electrodes.

In comparison, Figure 8a and 8b illustrate the size variation of the conventional PVA and porous PVA GPEs soaked in 6 m KOH electrolyte, which illustrates a smaller size expansion than the PAM matrix. Figure S8c and S8d (Supporting Information) further indicate that the pure PVA can absorb 0.34 g g^−1^ of KOH solution and 0.74 g g^−1^ water, while the porous PVA, with the enlarged interface by its porous structure, can uptake 0.39 g g^−1^ of KOH solution and 1.66 g g^−1^ of water. It is easily seen that the PAM owns a much larger liquid uptake ratio than the conventional PVA matrix, the incorporation with mSiO_2_ further improves the capacity.

In terms of electrolyte retention, Figure 4e illustrates the desorption rates of the electrolyte‐saturated GPEs exposed to ambient air for 72 h. The results indicate that the remaining weight of the pure PAM is 45.6% after 72 h, while the 5 wt.% mPAM remains 81.9%, and the 1 wt.% mPAM and 9 wt.% mPAM are in the middle of 75.1% and 78.8%, respectively. In comparison, Figure S9 (Supporting Information) displays that the weight of the pure PVA just remains 55.8% after 72 h, while the porous PVA is 70.9%. The electrolyte absorbed in the PAM is more easily to lose than the conventional PVA, but the incorporation with mSiO_2_ significantly improves the electrolyte retention.

Ionic conductivity is in close association with the ability of electrolyte uptake and retention. Figure 4f shows the trends of ion conductivity for the PAM‐based GPEs over 72 h. The ionic conductivity values gradually drop down from 97, 142, 322, and 204 mS cm^−1^ of the freshly pure PAM, 1 wt.% mPAM, 5 wt.% mPAM and 9 wt.% mPAM prepared GPEs, to 32, 67, 166, and 89 mS cm^−1^ of the ones after 72 h prepared GPEs. In comparison, Figure S10 (Supporting Information) displays that the ion conductivity of PVA also decreases rapidly after 72 h, from 85 and 110 mS cm^−1^ of the freshly preprepared pure PVA and porous PVA to of 12 mS cm^−1^ and 35 mS cm^−1^ after 72 h, respectively. Additionally, Table 2 presents a comparison of the performance between the PVA and PAM matrices, clearly highlighting the superior performance of the PAM material.

The above results show that the PAM owns significant advantage over the PVA, and the incorporation of mSiO_2_ nanoparticles further enhances the electrolyte uptake and retention as well as the ionic conductivity in a significant extent.

The performance of the mPAM GPEs is associated with the surface property of mSiO_2_ fillers. The as‐synthesized mSiO_2_ features a uniform mesoporous structure (≈3.6 nm in average diameter). Hwang et al investigated the adsorption and desorption of water in mesoporous silica with different pore sizes, where the capillary condensation and capillary evaporation for water uptake were elucidated.^[^ ^38^ ^]^ The ion transfer in the mSiO_2_ was also investigated by Fan^[^ ^39^ ^]^ and Ochs^[^ ^40^ ^]^ et al, where the surface‐charge‐mediated transport is dominated, in association with the surface properties (such as hydroxyl and silanol groups) on the pore walls. Therefore, the superior electrolyte uptake and retention, as well as ionic conductivity of the mPAM GPEs can be ascribed to the incorporation with mSiO_2,_ where the hydrophilic surface and internal microchannels can increase the electrolyte uptake and retention by capillary effect and hydrogen bond between the surface hydroxyl group and water molecule. Thereby, the ionic conductivity is significantly reduced due to the decreased crystallinity of PAM matrix and the enhanced ion migration by both surface and internal microchannel pathways provided by the continuous mSiO_2_ network across the membrane. The reinforcement of mSiO_2_ also optimizes the dimensional stability and homogenizes the current distribution when used in FZABs, where the charge–discharge current at the anode/GPE interface is more uniform than the pure PAM membrane, and the ORR/OER polarizations are also alleviated owing to the increase of ionic conductivity. At the cathode/GPE interface, the presence of mSiO_2_ assists the electrolyte transfer to the Co_3_O_4_ catalyst and enhances the ORR and OER kinetics during battery cycling.

## 3 Conclusion

We demonstrate a facile strategy to synthesize composite mesoporous silica/polyacrylamide (mPAM) GPEs. The optimized 5 wt.% mPAM GPE exhibits a high ionic conductivity was up to 322 mS cm^−1^, the weight loss after exposing in air 72 h was less than 18%, owing to its excellent property of electrolyte uptake and retention. Investigations show that the PAM matrix owns superior electrolyte uptake and retention over the conventional PVA, and the incorporation of mSiO_2_ further improves the electrolyte retention and ionic conductivity. When used as a quasi‐solid‐electrolyte, the rechargeable FZAB with the 5 wt.% mPAM GPE exhibited high electrochemical performance and structural stability, where the peak power density was up to 162.8 mW cm^−2^, and the initial charge‐discharge potential gap was as low as 0.62 V, resulting in a long lifespan exceeding 110 h, showcasing the combination of high durability, cost‐effectiveness and easy production for practical applications. These findings provide important insights for future designs of high‐performance quasi‐liquid gels and lay the foundation for the development of efficient and stable innovative FZABs.

## Conflict of Interest

The authors declare no conflict of interest.

## Supporting information



Supporting Information

Supplemental video 1

## Data Availability

The data that support the findings of this study are available on request from the corresponding author. The data are not publicly available due to privacy or ethical restrictions.
