# Supplementary material for: Composite Gel Polymer Electrolyte for High‐Performance Flexible Zinc‐Air Batteries
Source: Small. 2024 Nov 13;21(2):2408015. doi: 10.1002/smll.202408015 (PMC11735867; doi:10.1002/smll.202408015)
Supplement: Supplementary file 1 — Supporting Information [file SMLL-21-2408015-s001.docx]

**Supporting Information**

**Composite Gel Polymer Electrolyte for High-Performance Flexible Zinc-Air Batteries**

Yifan Liu^a^, Denise Bildan^c^, Xiangqun Zhuge^a^, Tong Liu^a^, Haoyang Zhong^d^, Zhihong Luo^b^, Hanhui Lei^c^, Kun Luo^a*^, Yurong Ren^a^, Maryam Bayati^c^ and Xiaoteng Liu^a,c*^

# Experimental

## 1.1 Materials and chemicals

Cetyltrimethylammonium bromide (CTAB, ≥99.0%), ethanol ( ≥99.7%), sodium hydroxide (NaOH, ≥96.0%), tetraethyl orthosilicate (TEOS, ≥28.4%), isopropanol (IPA, ≥99.7%), acetone (≥99.5%), carbon black (Vulcan XC-72), zinc acetate ((CH_3_COO)_2_Zn, ≥99.0) were purchased from Sino Pharm Reagent Co. Ltd. Potassium hydroxide (KOH, ≥90.0%), acrylamide (AM, MW~180-200), ammonium persulfate (APS), methylene bisacrylamide (MBA) were purchased from Shanghai Aladdin Biochemical Technology Co., Ltd. Polyvinyl alcohol (PVA, MW~19500), Polyethylene glycol (PEG, MW~20000), were bought from Shanghai Meryer Biochemical Technology Co., Ltd. Nano-tricobalt tetroxide (Co_3_O_4_, ≥99.5%), Zinc foil (0.3 mm), from Changsha Spring New Energy Technology Co., Ltd. Hydrophilic carbon cloth (WOS1002) was purchased from Kunshan Electronic Technology Co., Ltd. All aqueous solutions were prepared in deionised water (< 0.055 uS cm^-1^). All these drugs were of analytical purity and required no further treatment.

## 1.2 Preparation of mSiO_2_

The preparation process for mSiO_2_ refers to Supporting Information (SI) **S1a**. Initially, 0.3 g of CTAB, 25 mL of ETOH, 130 mL of DI water, and 0.15 g of NaOH are sequentially added into a 250 mL round-bottom flask. Subsequently, the flask is placed into a 35 ℃ oil bath and stirred magnetically at 650 rpm for 35 minutes. Then, 1 mL of TEOS is added, and the reaction is allowed to proceed for 6 h. Upon completion, the mixture is left to stand for 20 h. Sequential low-speed centrifugation is performed using DI water and ETOH, each for three cycles. Post-centrifugation, the sample is dried in a 60 ℃ oven for 12 h. The resulting white granules are ground finely, then subjected to calcination at 600 ℃ in a muffle furnace for 5 hours, yielding mSiO_2_ powder.

## 1.3 Preparation of mPAM GPEs

Synthesis of mSiO_2_ particles is shown in Supporting Information (Experimental 1.1 and **Fig.S1a**). **Fig.2a** depicts the preparation of the mPAM GPEs, where 0.1 g of mSiO_2_ powders were dispersed in 30 mL deionized water by vigorously stirring for 1 h, followed with the addition of 2 g of AM monomer, 10 mg of crosslinking agent MBA and 50 mg of initiator APS. The suspension was kept stirring for 1 h. Prior to polymerization, the above solution is degassed under nitrogen for 30 minutes. Then, polymerization is carried out at 60 °C for 5 h, led to the pure 5 wt.% mPAM GPE (ref. polymerization reaction in **Fig.S2**). After drying at 65 °C for 12 h, the mPAM was then immersed in the 6 mol L^-1^ KOH solution overnight, resulted in the 5 wt.% mPAM GPE with absorbed KOH solution.

As reference, 0.02 g, 0.18 g of mSiO_2_ powders were also used to prepare the GPEs, following the method described above, marking as 1 wt.% mPAM and 9 wt.% mPAM GPE, respectively. Moreover, the PVA-based GPEs were also fabricated to show the effect of matrix and porosity on the ionic conductivity (more details please see the Supporting Information).

## 1.4 Preparation of PVA GPEs

5 g of PVA powder was weighed into a beaker containing 40 mL and stirred at 90 °C and 450 rpm for 1.5 h. Then 1.5 g of PEG dissolved in appropriate amount of acetone was added dropwise to the solution and stirring was continued for 1 h to obtain a homogeneous transparent viscous mixture. The mixture was poured into a 10×10 cm square mould, cooled at room temperature for a period and then put into the -3 °C freezing crosslinking for 10 h. After the crosslinking, the resulting polymer electrolyte was soaked in ETOH at room temperature for 20 h, in order to dissolve the PEG and obtain the dried polymer film. Subsequently, the dried membrane was soaked in 6 M KOH for 8 h to obtain porous PVA hydrogels.

Pure PVA hydrogels were synthesized by the same method without the addition of PEG.

## 1.5 Experimental testing and characterization

The absorption of deionized water and KOH electrolyte was measured and analyzed using a precision electronic balance (LD523/E). Samples immersed in a sufficient amount of solution were periodically weighed at intervals, under conditions of 24°C and 50% relative humidity. Prior to weighing, the surface of the hydrogel was wiped with lint-free paper to minimize the influence of surface electrolyte on the experimental results. Similarly, desorption of KOH was analyzed using the precision electronic balance, with samples exposed to air tested once every day.

Absorption of deionised water, KOH electrolyte was analysed using a precision electronic balance at room temperature on samples soaked in a sufficient amount of solution recorded at hourly intervals, the surface of the hydrogel was wiped with a dust-free paper prior to weighing in order to minimise the effect of the surface electrolyte on the experiment. Desorbed KOH was also analysed using a precision electronic balance. The rates of uptake (Mt) and desorption (M't) of electrolytes by GPEs were calculated according to the formulae ^[24]^.

$M_{t}= \frac{M_{1}-M_{0}}{M_{0}}$（1）

$${M'}_{t}= \frac{M_{3}}{M_{2}} （2）$$

*M_0_:* mass of dried GPE; *M_1_:* mass of GPE after absorption of electrolyte; *M_2_:* mass of GPE saturated by absorption in electrolyte solution; *M_3_:* mass of GPE after desorption of electrolyte.

The electrochemical impedance spectroscopy (EIS) test was carried out on electrochemical workstation CHI 660, setting the test mode to AC impedance mode, operating frequency of 0.1 - 100 kHz, voltage amplitude of 5 mV, and the formula for calculating ionic conductivity:

$$\sigma=\frac{l}{ESR*A}$$

Where *l, A* represent the thickness of the polymer electrolyte involved in the test, the contact area between the electrolyte and the stainless-steel sheet, respectively, while the value of *ESR* can be obtained from the intersection of the image in the AC impedance spectrum with the real axis.

Tensile-strain tests were performed using an electronic universal testing machine-10 kN (WDT-1010 KN) with a tensile spacing of 20 mm and a tensile rate of 50 mm min^-1^.

## 1.6 Assembly of flexible zinc-air batteries

The carbon cloth was firstly cut into pieces in size of 1.5 × 2 cm, which were rinsed with acetone, ethanol and deionized water in sequence, and then was dried in an oven at 60 ℃. A catalyst ink was prepared by mixing 27 mg of Co_3_O_4_, 63 mg of carbon black and 0.9 mL of Nafion solution into a mixture of 7.2 mL deionized water and 1.8 mL of IPA by ultrasonication for 30 min. The ink was sprayed on the pretreated carbon cloth with a mass loading of 0.3 mg cm^-2^, and dried in an oven at 60 °C.

In this study, FZABs were assembled in a "sandwich" configuration. The setup included a polished zinc foil as the negative electrode to eliminate the oxide layer, followed by a GPE, and a catalyst-loaded air electrode at the positive side. To enhance the stability of the cell structure, breathable bandages were applied. Battery testing was performed using a Wuhan LAND multi-channel test system (LAND CT2001A). For charge/discharge tests, the cut-off voltage ranged from 0.5 V to 2.5 V, with a constant current density of 3 mA cm^-2^ for both charge and discharge, each lasting 10 minutes. Full discharge tests maintained the same current density at 3 mA cm^-2^, with a cut-off voltage of 0.6 V. Rate performance was evaluated through variable discharges at 0.5, 1, 3, 5, 7, and 9 mA cm^-2^. Additionally, polarization curves were recorded using an electro-workstation set at a scan rate of 20 mA s^-1^.

## 1.7 Characterizations

The morphology and structure of the freeze-dried mPAM GPEs were characterized by SEM (SEU8010) and EDX energy spectroscopy. Small angle XRD analysis was used to investigate the mSiO_2_ using Bruker D8 Advance diffractometer (Cu Kα). Moreover, FTIR (Thermo Scientific iS5d FT-IR) and Brunauer-Emmett-Teller (BET, Micromeritics ASAP 2460) method were also employed to identify the composition and pore structure of mSiO_2_ particles. Other performance testing details are described in the Supporting Information.

## 1.8 Statistical Analysis

Statistical Analysis: To characterise the materials, techniques including SEM, XRD, TEM, FTIR, and BET surface area analysis were employed. GPE samples were randomly selected after in-situ polymerisation and cut for testing. Absorption and storage experiments with H_2_O and KOH were conducted under controlled conditions (25°C and 55% RH), with each test repeated at least five times. The results for electrolyte absorption, storage capacity, and ionic conductivity are presented as mean values with standard deviations. Battery performance tests were also conducted at 25°C and 55% RH, and the results were averaged from at least four flexible zinc-air battery assemblies. Data visualisation was performed using Microsoft Excel.

# Supplementary Figures


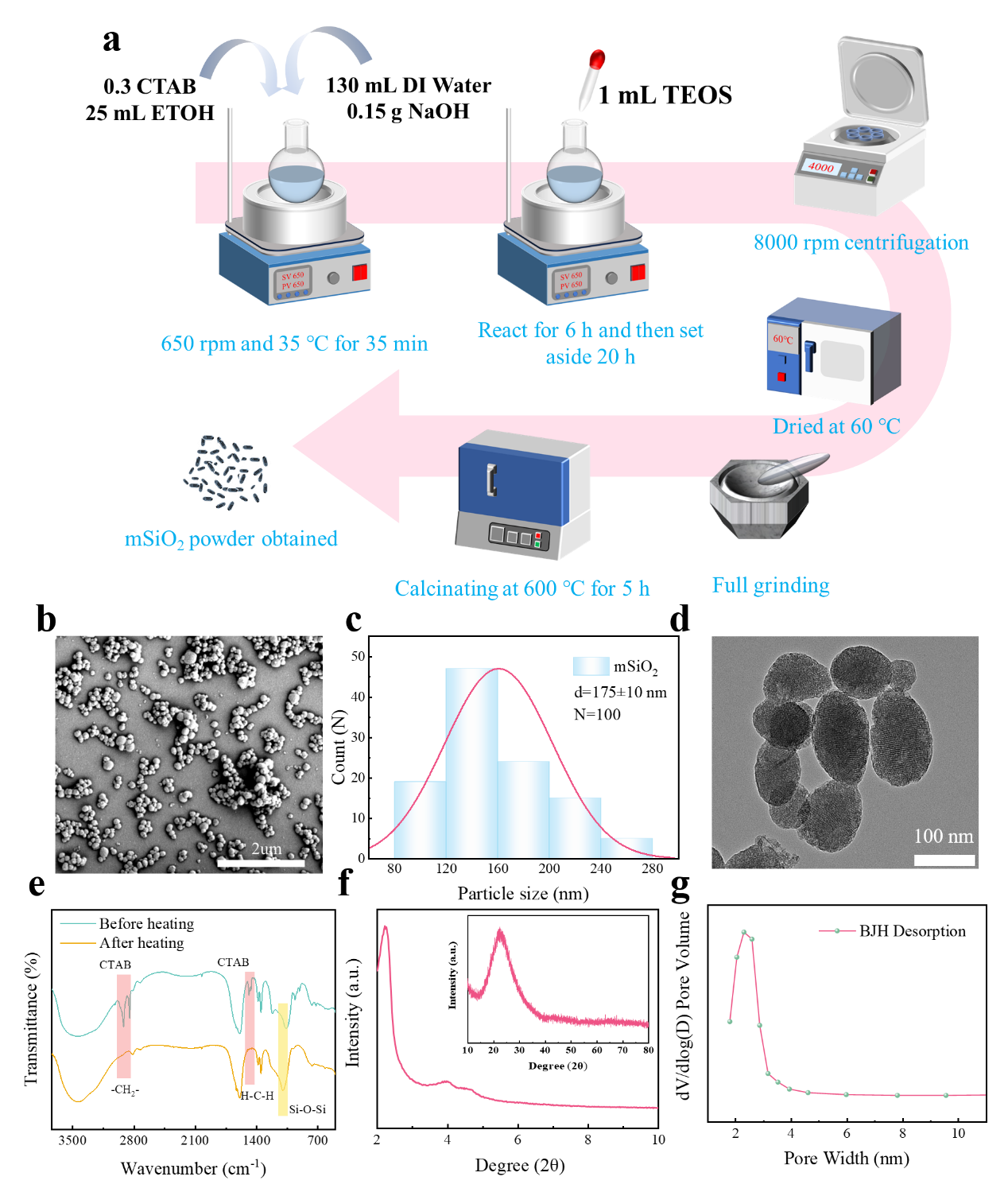


**Fig. S1** Synthesis of mSiO_2_ nanoparticle (a); SEM (b), size distribution (c) and HRTEM (d) images of mSiO_2_ particles; FTIR (e) of the SiO_2_ gel (before calcination) and mSiO_2_ (after calcination); XRD (f) and BET (g) analyses of mSiO_2_

**
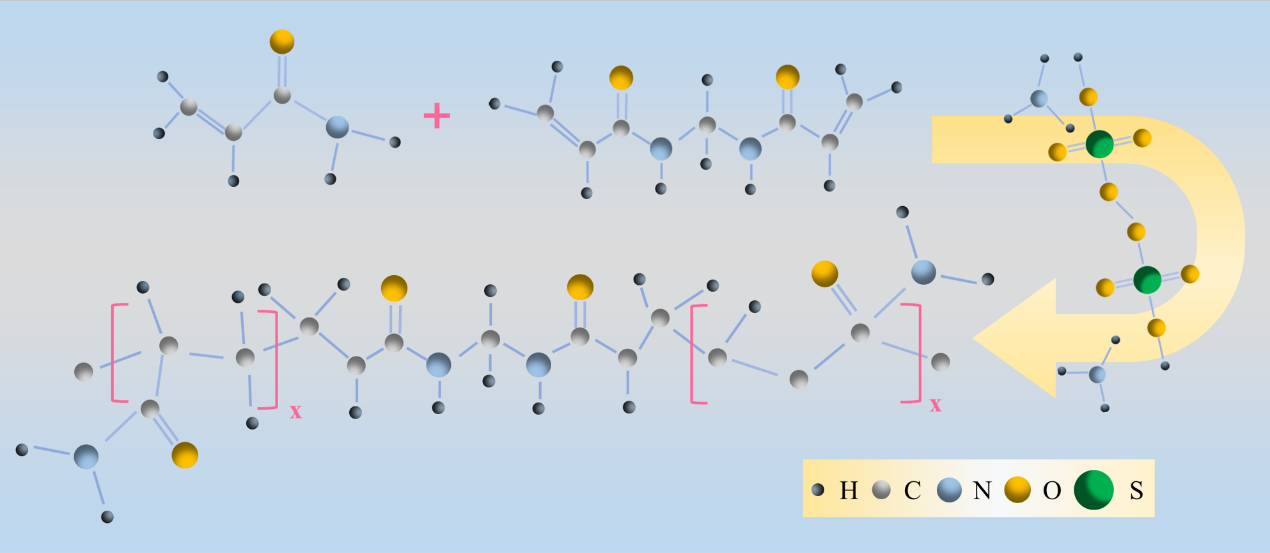
**

**Fig. S2** Schematic diagram of PAM synthesis


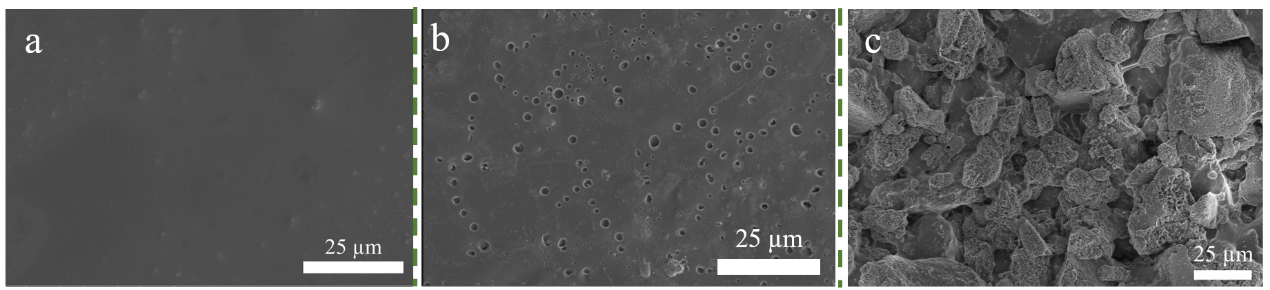


**Fig. S3** SEM images of the pure PAM (a), 1 wt.% mPAM (b) and 9 wt.% mPAM (c) GPEs


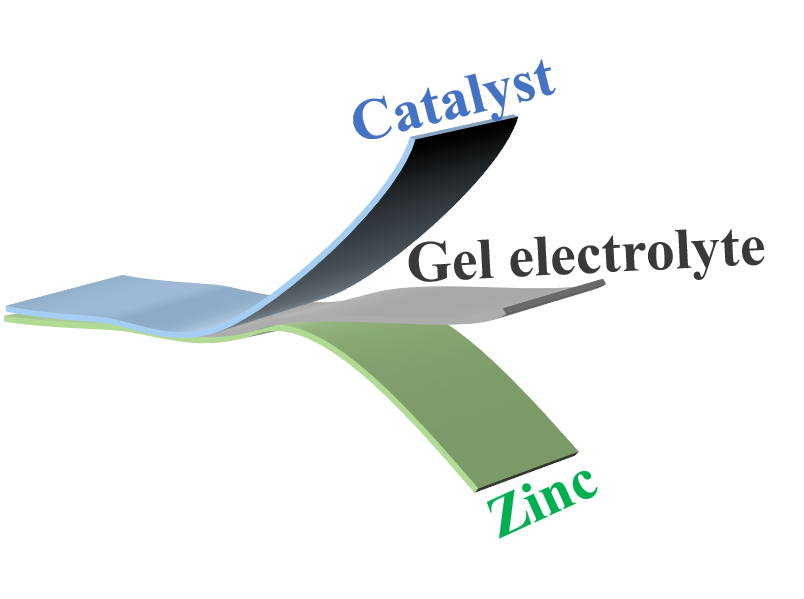


**Fig. S4** Schematic structure of FZAB


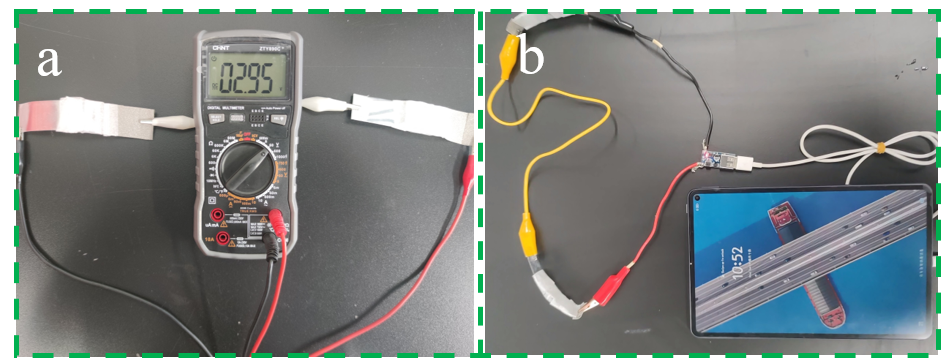


**Fig. S5** Measurement of the open-circuit potential for a two-FZAB pack in series (a); charging a tablet for 1 h with the two-FZAB pack.


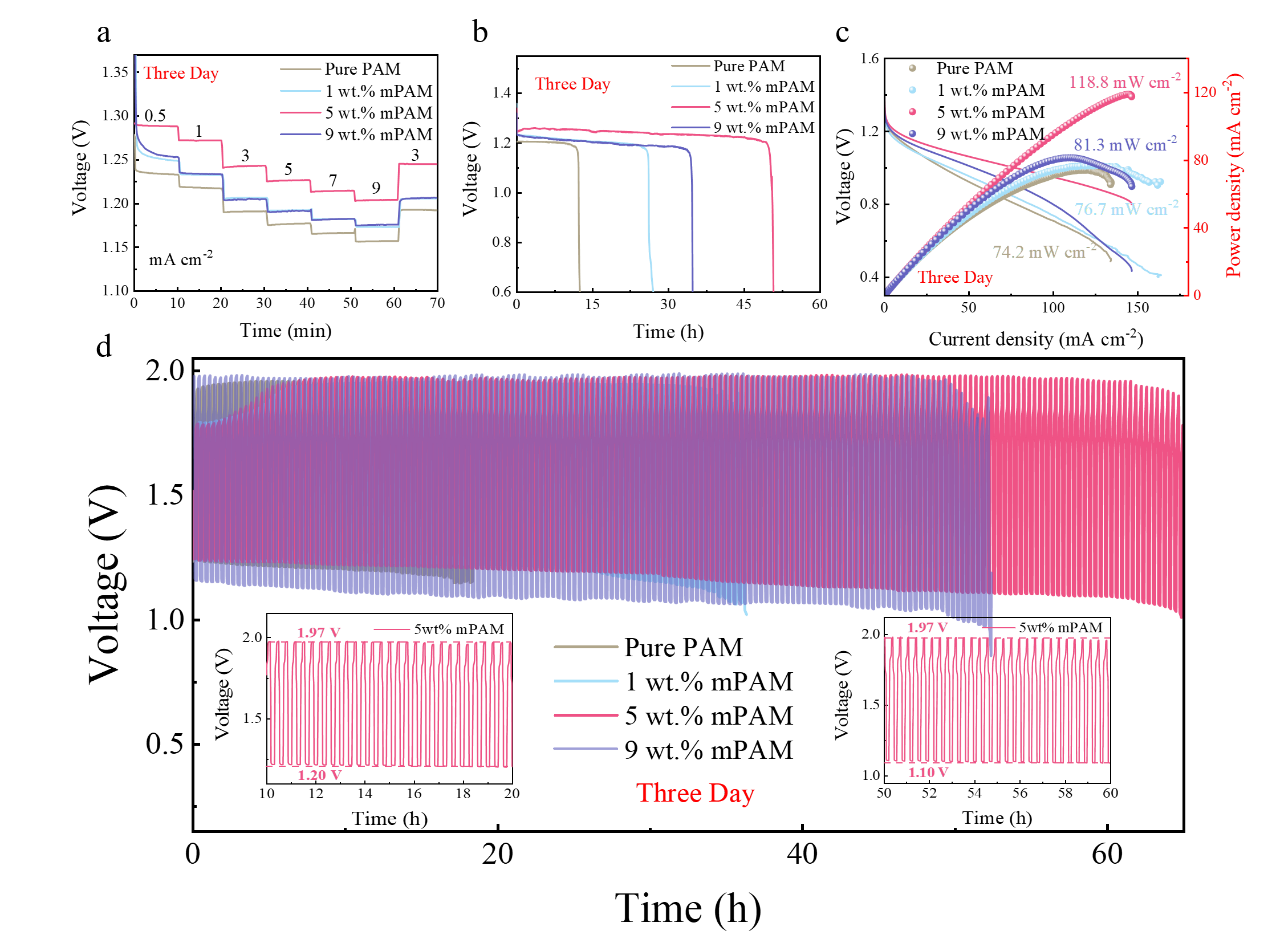


**Fig. S6** Rate performance (a), full-discharge capacity at 3 mA cm^-2^ (b), discharge polarization and power density curves (c), and cyclability at 3 mA cm^-2^ (d) of the FZABs with the pure PAM, 1 wt.% mPAM, 5 wt.% mPAM and 9 wt.% mPAM GPEs after three days (72 h), the inset highlights the cycles with the 5 wt.% mPAM GPE at the 10~20 h and 90~100 h intervals.


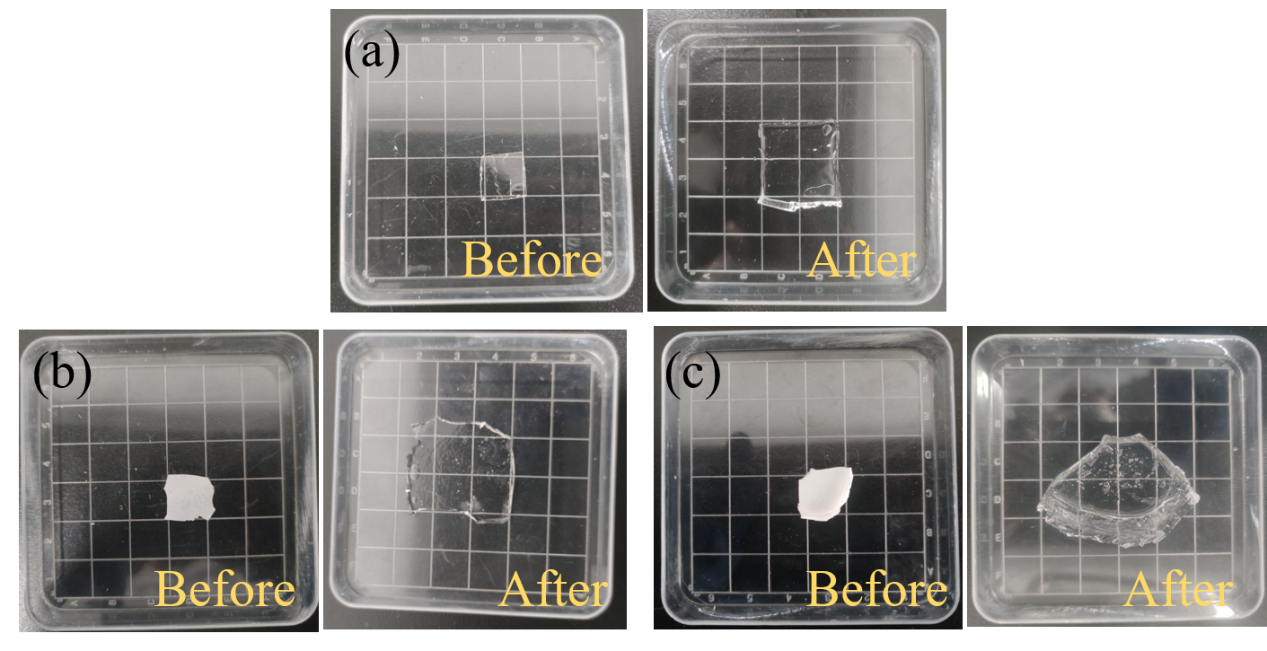


**Fig. S7** Swelling of the pure PAM (a), 1 wt.% mPAM (b) and 9 wt.% mPAM (c) GPEs


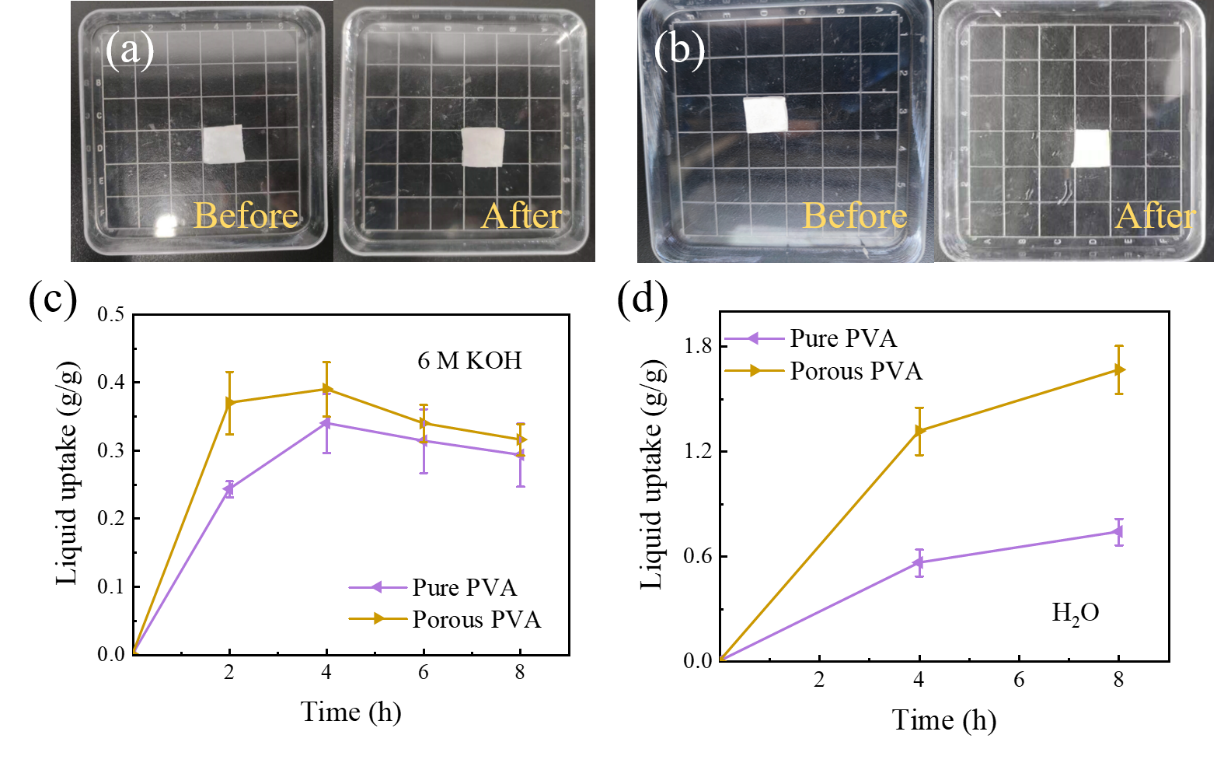


**Fig. S8** Photos of the pure PVA (b) and porous PVA (b) GPEs before and after swelling in KOH electrolyte; liquid uptake ratios of 6 mol L^-1^ KOH (c) and deionized water (d) in the pure PVA and porous PVA GPEs

**Fig.S8a** and **S8b** illustrate the changes in appearance and size for the pure PVA and porous PVA after absorbing 6 M KOH electrolyte, showing minimal variation in morphology size before and after saturation. **Fig. S8c and S8d** illustrate the uptake capacity of the pure PVA and porous PVA GPE, where the former can uptake 0.34 g g^-1^ of KOH solution and 0.74 g g^-1^ water, while the latter, with its porous structure, can uptake 0.39 g g^-1^ of KOH solution and 1.66 g g^-1^ of water.


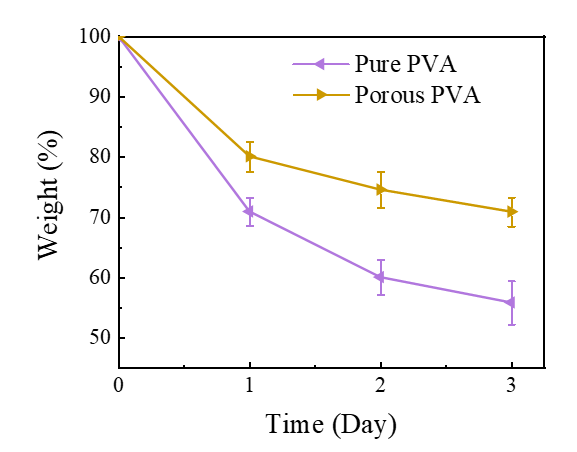


**Fig. S9** Water retention of the pure PVA and porous PVA GPEs

As shown in **Fig.S9**, after 3 days, the desorption rate of the pure PVA is 55.8%, while the porous PVA demonstrates significantly better electrolyte retention (70.9%) compared to the pure PVA.


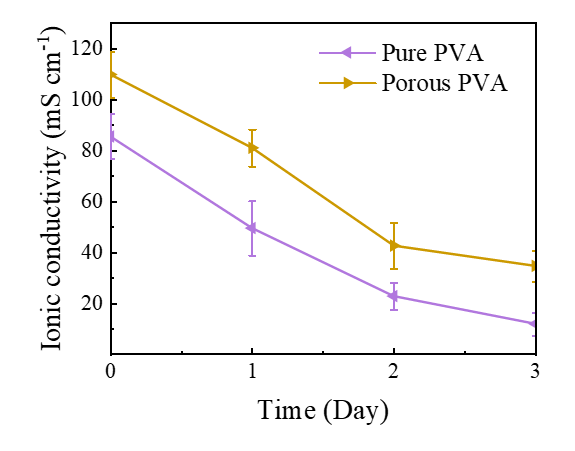


**Fig. S10** Changes in ionic conductivity of the pure PVA and porous PVA over three days

**Fig.S10** shows the changes in ion conductivity of the pure PVA and porous PVA. The ionic conductivity of the PVA based GPE decreases rapidly. After three days, the ionic conductivity of the porous PVA went down to 34.7 mS cm^-1^, while the pure PVA exhibited an ion conductivity of 11.9 mS cm^-1^ similarly after three days.


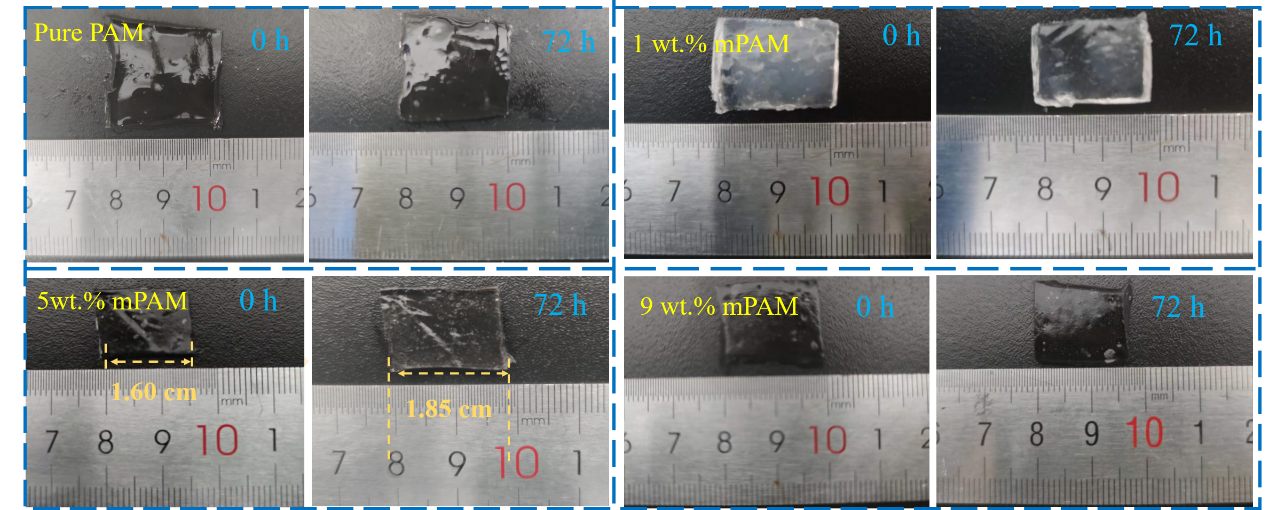


**Fig. S11** Dimensional stability after 72 h for the pure PAM, 1 wt.% mPAM, 5 wt.% mPAM and 9 wt.% mPAM GPEs


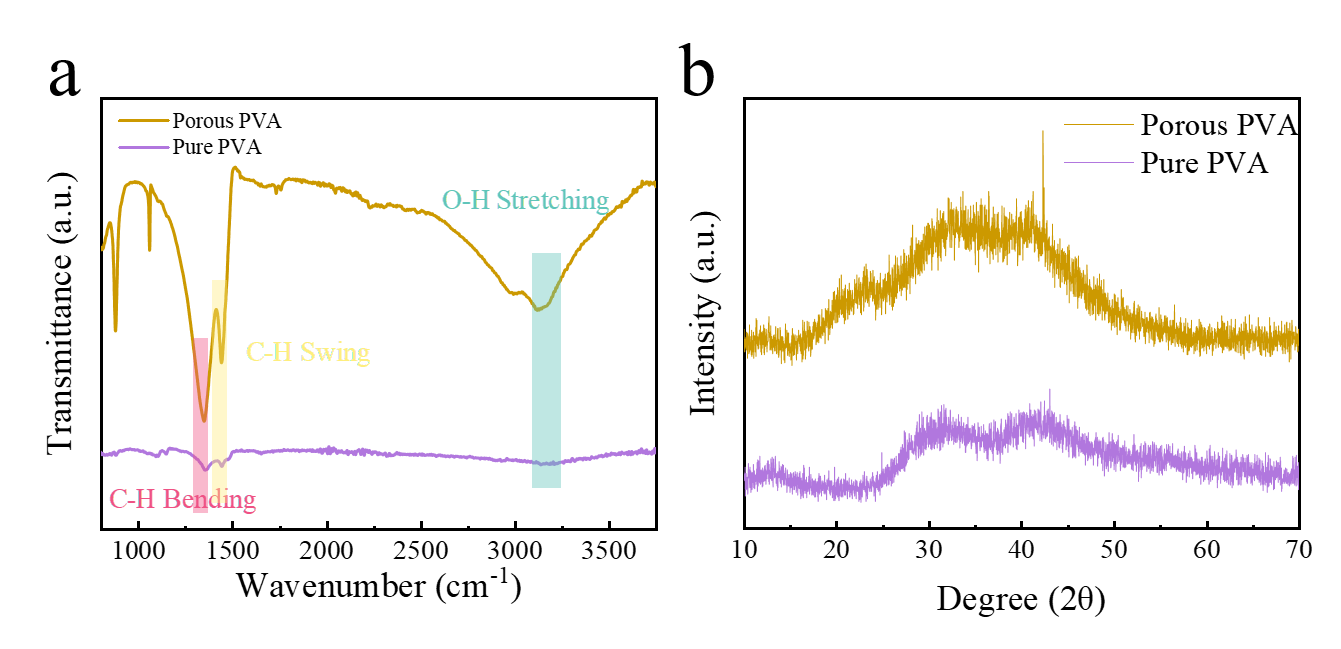


**Fig. S12** FTIR (a) and XRD (b) spectra of the pure PVA and porous PVA GPEs

**Fig.S12a** a broad absorption peak in the range of 3100-3400 cm^-1^, primarily attributed to the stretching vibrations of O-H bonds. The absorption peaks at 1443 cm^-1^ and 1357 cm^-1^ are associated with C-H bending and C-H wagging, respectively. Compared to pure PVA, the porous PVA presents much higher peak at 3160 attributed to O-H bond, indicating larger water retention in agreement with previous literature ^[1]^. XRD analysis shown in **Fig.S12b** illustrates that pure PVA displays an amorphous structure with two broad peaks at 2θ values of 33° and 42°. In comparison, the peaks for Porous PVA are even broader, indicating its lower crystallinity.


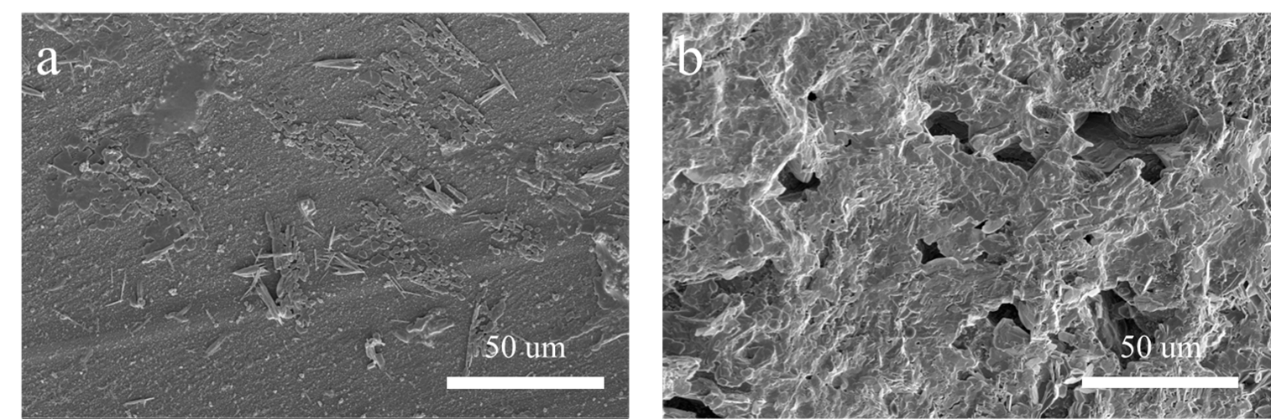


**Fig. S13** SEM images of the pure PVA (a) and porous PVA (b) GPEs

SEM images of the pure PVA and porous PVA GPEs as shown in **Fig.S13**. **Fig.S13a** reveals that the surface of the pure PVA is flat, and in **Fig.S13b** there are irregular pores on the surface of the porous PVA, which are formed by dissolution of the pore-forming agent PEG in ethanol and evaporation.


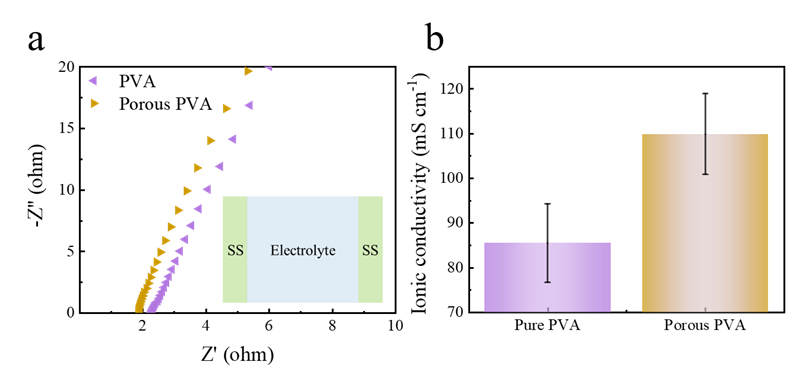


**Fig. S14** (a) AC impedance plots and x-axis intersection magnification of Pure PVA and Porous PVA GPEs; (b) comparison of ionic conductivity.

In **Fig.S14a**, the impedance value of Porous PVA (1.83 Ω) is lower than that of Pure PVA (2.31 Ω), and the corresponding ionic conductivity of Porous PVA (109.8 mS cm^-1^) in **Fig.S14b** is also higher than that of Pure PVA (85.4 mS cm^-1^).


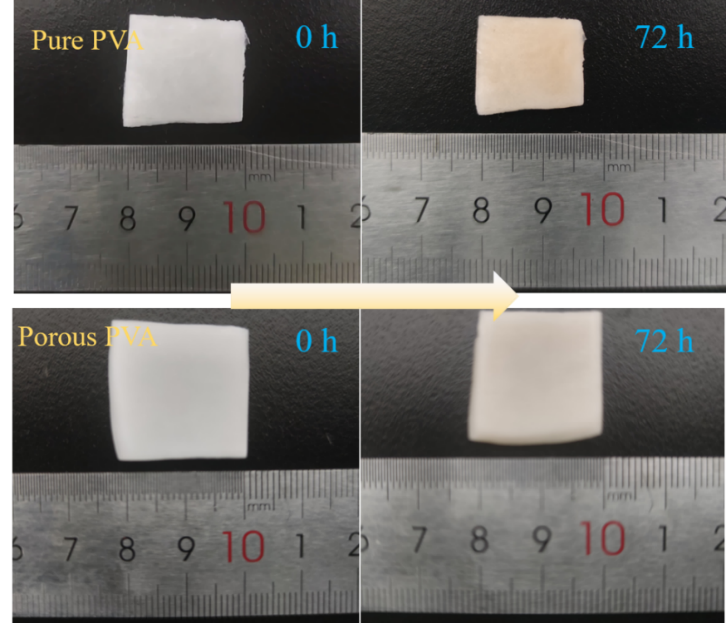


**Fig. S15** Dimensional changes at 72 h for (a) Pure PVA, (b) Porous PVA GPEs.

**Fig.S15** presents a comparison of size stability between Pure PVA and Porous PVA, showing that the length of Pure PVA decreases by approximately 0.30 cm after 72 h, while the size and morphology of Porous PVA remain almost unchanged. This further demonstrates that a porous structure contributes to the stability of hydrogel size.


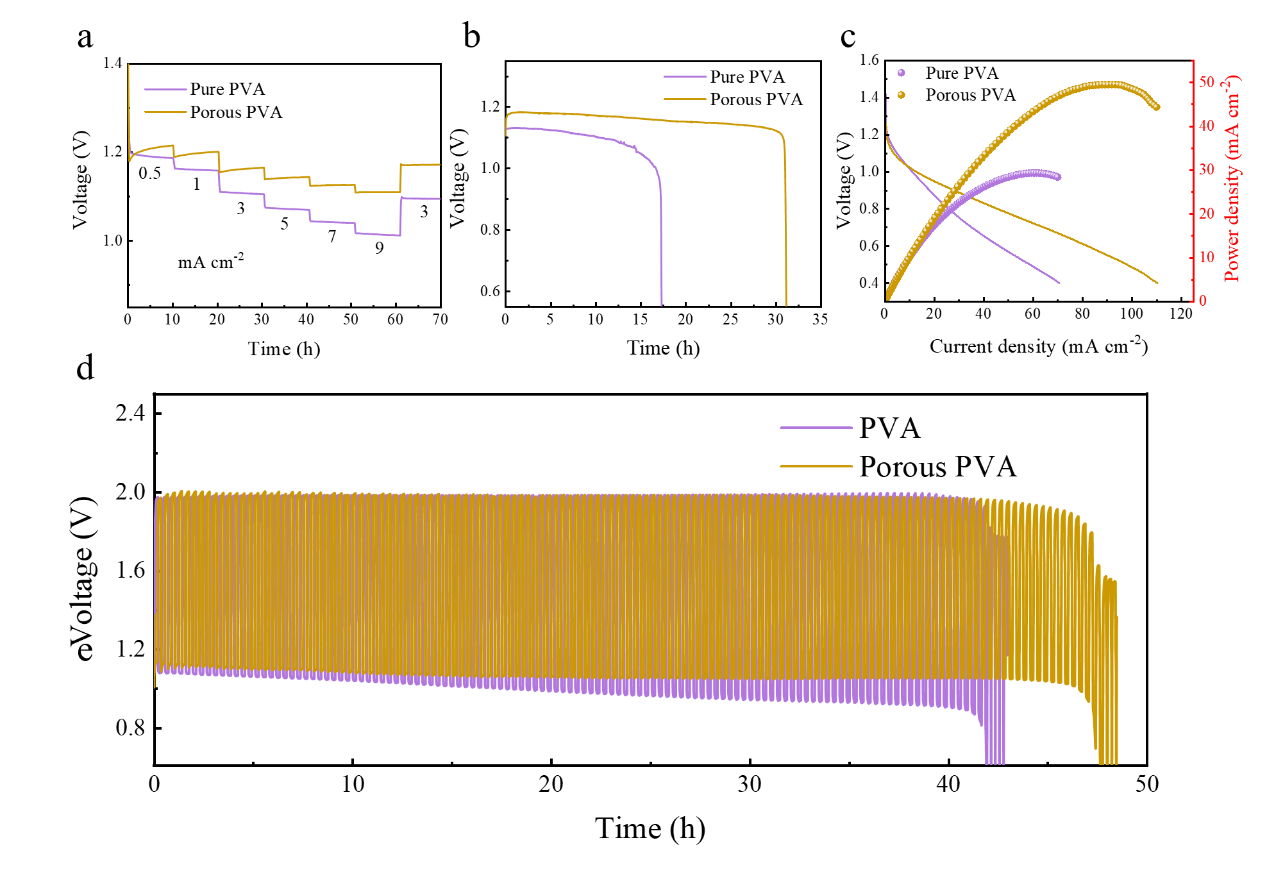


**Fig. S16** Rate performance (a), full-discharge capacity at 3 mA cm^-2^ (b), discharge polarization and power density curves (c), and cyclability at 3 mA cm^-2^ (d) of the FZABs with the pure PVA and porous PVA GPEs

**Fig.S16a** illustrates the discharge rate performance of the pure PVA and porous PVA, with the latter showing a 0.11 V decrease in discharge platform, surpassing the pure PVA. In **Fig.S16b**, the discharge duration of the porous PVA (32 h) exceeds that of the pure PVA (17 h). **Fig.S16c** shows the polarized power density profiles of the FZABs with the pure PVA and porous PVA GPEs, where the porous PVA GPE achieved a power density of 49.6 mW cm^-2^ at a current density of 90.1 mA cm^-2^. In contrast, the pure PVA achieved a power density of 29.4 mW cm^-2^ at a current density of 59.4 mA cm^-2^. **Fig.S16d** demonstrates the longer charge-discharge performance, the porous PVA reaches 46 h, surpassing the pure PVA of 41 h.


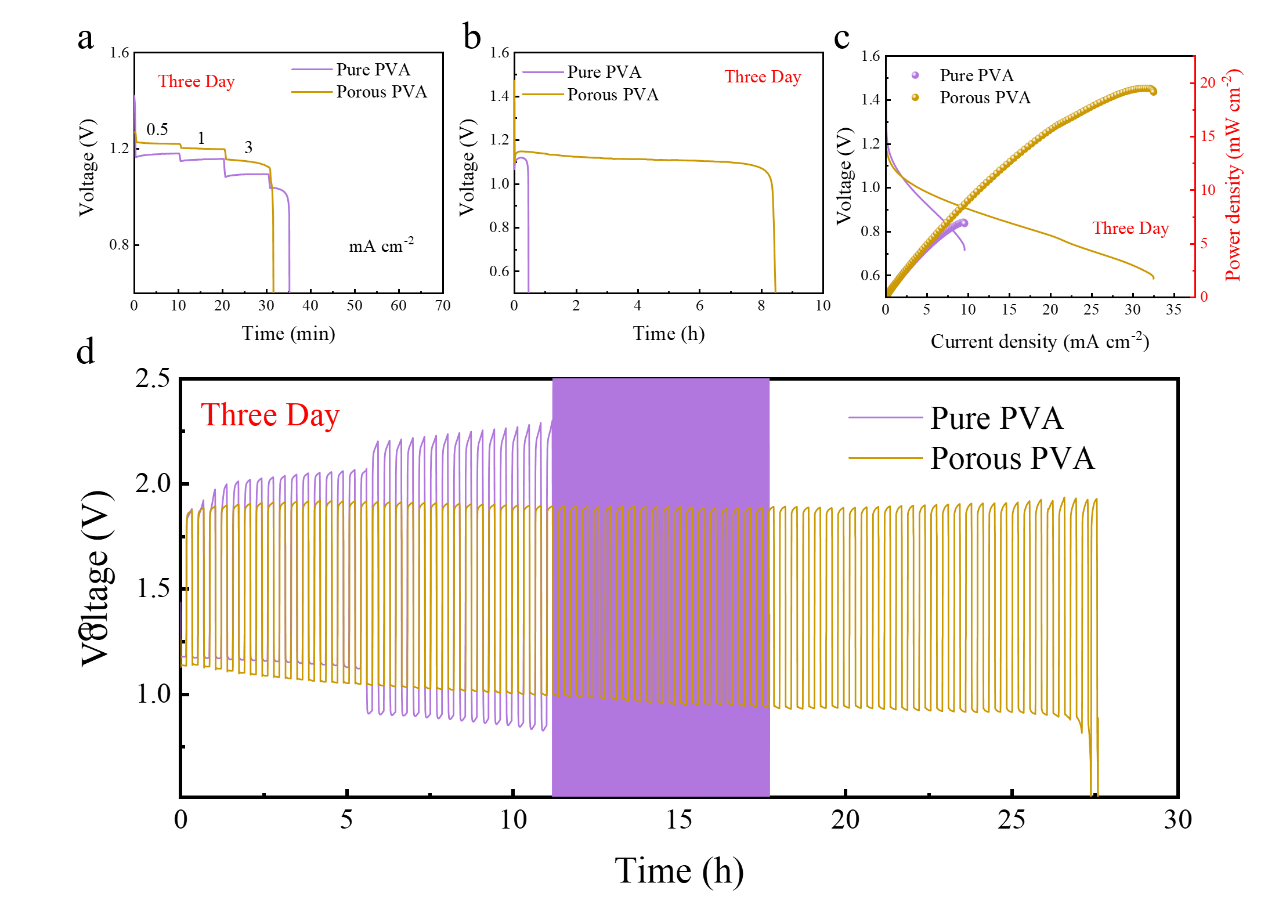


**Fig. S17** Rate performance (a), full-discharge capacity at 3 mA cm^-2^ (b), discharge polarization and power density curves (c), and cyclability at 3 mA cm^-2^ (d) of the FZABs with the pure PVA and porous PVA GPEs after three days (72 h)

**Fig.S17a** illustrates the discharge rate performance of the pure PVA and porous PVA. Unfortunately, the batteries could no longer discharge normally when the current density reached 5 mA cm^-2^, indicating that the pure PVA and porous PVA after three days were unable to operate properly under high current density. In **Fig.S17b**, the discharge time with the porous PVA GPE after three days was 8 h, while the value for the pure PVA decays rapidly, lasting less than 1 h. The power density of the porous PVA GPE is 19.4 mW cm^-2^ at a current density of 31.3 mA cm^-2^ (see **Fig.S17c**). For the pure PVA, the power density is 7.1 mW cm^-2^ at a current density of 9.4 mA cm^-2^. **Fig.S17d** demonstrates that the FZAB with the porous PVA exhibits a discharge performance of 27 h, while the cyclic lifespan with the pure PVA is only 12 h.

# Supplementary Tables

**Table S1** Performance comparison with the reported gel polymer electrolytes for flexible zinc-air batteries

| **Electrolyte** | **Power Density**  **(mW cm^-2^)** | **Ionic conductivity**  **(mS cm^-1^)** | **Cycling Stability (current density)** | **Ref.** |
| --- | --- | --- | --- | --- |
| Porous PVA-KOH | 62.6 | 57.3 | 48 h  (2 mA cm^-2^) | [1] |
| PAM-F/G-KOH | 155 | 276 | 40 h  (2 mA cm^-2^) | [2] |
| PAAK-M-KOH | 126 | 289 | 55 h  (2 mA cm^-2^) | [3] |
| PAANa-KOH | 100.7 | 190 | 60 h  (10 mA cm^-2^) | [4] |
| p18@PAA-KOH | 154.5 | 562.6 | 27 h  (2 mA cm^-2^) | [5] |
| PAM-KOH | 39 | 330 | 55 h  (2 mA cm^-2^) | [6] |
| PVA-KOH | 50.5 | 30 | 13 h  (3 mA cm^-2^) | [7] |
| PVA-GTAC-NH_4_Cl-KOH | 8 | 48.3 | 40 h  (1 mA cm^-2^) | [8] |
| PAA-ZnO-KOH | 54.6 | / | ＞40 h  (1 mA cm^-2^) | [9] |
| PVA–KOH | 21 | 16 | ＞10 h  (2 mA cm^-2^) | [10] |
| QA-NC/GO-KOH | 50 | 39.0 | ＞10 h  (1 mA cm^-2^) | [11] |
| Neutral GPE | / | 76.1 | 70 h  (1 mA cm^-2^) | [12] |
| Starch gel-KOH | 80 | 111.5 | ＞35 h  (2 mA cm^-2^) | [13] |
| PAA-KC | 106.8 | 165.4 | ＞60 h  (2 mA cm^-2^) | [14] |
| PVA-CMC based GPES | / | 79 | 104 h  (5.2 mA cm^-2^) | [15] |
| PANa hydrogel | 144.6 | 81.2 | ＞70 h  (2 mA cm^-2^) | [16] |
| PAM-SC | 107.7 | 324.6 | ＞400 cycles  (0.5 mA cm^-2^) | [17] |
| 5wt% mPAM | 162.8 | 322 | 115 h  (3 mA cm^–2^) | This work |

**Table S2** Comparison of the PVA and PAM-based GPEs in this article

| Items | Pure PAM | 1 wt.% PAM | 5 wt.% PAM | 9 wt.% PAM | Pure PVA | Porous PVA |
| --- | --- | --- | --- | --- | --- | --- |
| KOH uptake  (g g^-1^) | 22 | 25 | 37 | 28 | 0.34 | 0.39 |
| H_2_O uptake  (g g^-1^) | 17 | 20 | 30 | 23 | 0.74 | 1.66 |
| Desorption rates  (%) | 45.6 | 75.1 | 81.9 | 78.8 | 55.8 | 70.9 |
| Ionic conductivity  (mS cm^-1^) | 97 | 143 | 322 | 204 | 85 | 110 |
| Discharge performance  (h) | 37 | 46 | 75 | 47 | 17 | 32 |
| Power density  (mW cm^-2^) | 109.7 | 114.9 | 162.8 | 125.1 | 59.4 | 90.1 |
| Cyclic performance  (h) | 56 | 77 | 115 | 67 | 41 | 46 |

# Reference

1. X. Y. Fan, J. Liu, Z. S. Song, X. P. Han, Y. D. Deng, C. Zhong, W. B. Hu, *Nano energy* **2019**, *56*, 454.
2. P. F. Zhang, K. L. Wang, Y. Y. Zuo, M. H. Wei, H. W. Wang, Z. Chen, N. Shang, P. C. Pei, *ACS Appl. Mater. Interfaces* **2022,** *14*, 49109.
3. P. F. Zhang, K. L. Wang, Y. Y. Zuo, M. H. Wei, H. W. Wang, Z. Chen, N. Shang, P.C. Pei, *Chem. Eng. J.* **2023**, *451*, 138622.
4. S. Y. Zhao, K. L. Wang, S. X. Tang, X. T. Liu, K. L. Peng, Y. Xiao, Y. Chen, *Energy Technol.* **2020**, *8*, 1901229.
5. N. Shang, K. L. Wang, M. H. Wei, Y. Y. Zuo, P. F. Zhang, H. W. Wang, Z. Chen, D. Y. Zhong, P. C. Pei, *Adv. Funct. Mater.* **2023**, *33*, 2303719.
6. M. -J. Tan, B. Li, P.L. Chee, X.M. Ge, Z.L. Liu, Y. Zong, X. -J. Loh, *J. Power Sources* **2018**, *400*, 566.
7. X. Y. Fan, J. Liu, J. Ding, Y. D. Deng, X. P. Han, W. B. Hu, C. Zhong, *Fron. Chem.* **2019**, *7*, 678.
8. C. Lin, S. S. Shinde, X. P. Li, D. H. Kim, N. W. Li, Y. Sun, X. K. Song, H. J. Zhang, C. H. Lee, S. U. Lee, J. H. Lee, *ChemSusChem* **2018**, *11*, 3215.
9. X.X Shu, S. Chen, S. Chen, W. Pan, J.T. Zhang, *Carbon* **2020**, *157*, 234.
10. X. Chen, B. Liu, C. Zhong, Z. Liu, J. Liu, L. Ma, Y. Deng, X. Han, T. Wu, W. Hu, J. Lu, *Adv. Energy Mater.* **2017**, *7*, 1700779.
11. J. Zhang, J. Fu, X. Song, G. Jiang, H. Zarrin, P. Xu, K. Li, A. Yu, Z. Chen, *Adv. Energy Mater.* **2016**, *6*, 1600476.
12. Y. Li, X.Y. Fan, X. R. Liu, S.X. Qu, J. Liu, J Ding, X. P. Han, Y.D. Deng, W. B. Hu, C. Zhong, *J. Mater. Chem. A* **2019**, *7*, 25449.
13. M. Li, B. Liu, X. Y. Fan, X. R. Liu, J. Liu, J. Ding, X. P. Han, Y. D. Deng, W. B. Hu, C. Zhong, *Cell Rep. Phys. Sci.* **2022**, *3*, 100687.
14. H. W. Wang, K. L. Wang, R. Zhang, B. Liang, Z. Chen, M. H. Wei, J. Y. Xiong, D. Y. Zhong, Y. Y. Zuo, P. C. Pei, *Chem. Eng. J.* **2024**, *498*, 155539.
15. D. Choudhary, R. Bala, R. Dhiman, *J. Electrochem. Soc.* **2024,** *171*, 070533.
16. Z. Pei, Y. Huang, Z. Tang, L. Ma, Z. Liu, Q. Xue, Z. Wang, H. Li, Y. Chen, and C. Zhi, *Energy Storage Mater*. **2019**, *20*, 234.
17. M. L. Jiao, L. X. Dai, H. R. Ren, M. T. Zhang, X. Xiao, B. Wang, J. L. Yang, B. L. Liu, G. M. Zhou, H. M. Cheng, *Angew. Chem.* **2023**, *135*, e202301114.
